# Support for market-based and command-and-control congestion relief policies in Latin American cities: Effects of mobility, environmental health, and city-level factors

**DOI:** 10.1016/j.tra.2020.12.004

**Published:** 2021-02-26

**Authors:** Xize Wang, Daniel A. Rodríguez, Anjali Mahendra

**Affiliations:** a Department of Real Estate, National University of Singapore, Singapore; b Department of City and Regional Planning, University of California, Berkeley, USA; c World Resources Institute, WRI Ross Center for Sustainable Cities, Washington, D.C., USA

**Keywords:** Congestion pricing, Traffic demand management, Driving restrictions, Public acceptance, Automobile regulation, Latin America

## Abstract

Public support for the implementation of congestion relief policies is critical for the policies’ technical and political success. To identify the personal, social, and city-level factors associated with higher acceptance towards such policies, this study uses a 2016 survey of 8178 residents from 11 cities across 10 Latin American countries collected by the Development Bank of Latin America (*Corporación Andina de Fomento* or CAF). We examined support for two demand-side approaches to managing the traffic congestion externality: congestion pricing – a market-based approach, and driving restrictions or bans – a command-and-control approach. Logit regression models show that personal mobility such as owning or using a private vehicle during a respondent’s main commute trip are associated with decreased support, while higher congestion delay in one’s commute and having a young child recently diagnosed with respiratory problems increases support for either congestion relief policy. In addition, residents of cities with higher levels of median annual particulate matter and with prior experience with traffic bans expressed higher support for either policy. Residents of cities with higher income inequality supported only driving restrictions; while those of cities with higher GDP per capita had lower support only for congestion pricing. To improve the public acceptance of congestion relief policies in Latin America, policy makers could: (1) explicitly seek to mitigate the costs it brings on individuals by investing in substitutes like public transportation; (2) promote the personal and social environmental and health benefits; (3) consider beginning with temporary, pilot programs; and in the case of driving restrictions, (4) take into account city-specific conditions related to income inequality that may influence public support for the policies.

## 1 Introduction

Vehicular congestion and air pollution continue to impose enormous economic costs on urban residents. For world cities, estimates of yearly congestion costs per driver are in the thousands of dollars (INRIX, 2019), with per km traveled cost estimates ranging between USD$0.1 (Bayliss and Glaister, 2011) and $0.26 (DfT, 2010). Conditions are similar, if not worse, in some large Latin American cities. For example, in Sao Paulo and Mexico City drivers spend more than 50 hours a year in peak traffic congestion (“The Hidden Cost”, 2018). In Bogotá, the cost of congestion has been estimated at 1% of the daily earned wage of workers (Akbar and Duranton, 2017). To manage the negative externality of congestion, policy makers typically adopt supply-side interventions such as expanding road and transit capacity. However, there is an increasing number of demand-side interventions being implemented, that attempt to better manage the demand for automobile travel, especially where road space is limited and difficult to expand. Policies to manage travel demand can be broadly classified as either market-based or command-and-control. Market-based policies commonly involve raising out-of-pocket costs of travelers by charging them a fee which is likely to reduce demand and bring the private marginal cost of travel more in line with social marginal cost. Fees can vary by time of day, as in the Electronic Road Pricing system in Singapore (Goh, 2002), can change dynamically in response to the real-time traffic condition, as in I-10 in Southern California (Bento et al., 2017), or can remain flat throughout the day as in London (Givoni, 2011). Command-and-control approaches harness the police power of the state to limit the circulation of vehicles either in the entire city or in subareas. Many cities such as Beijing, Bogota and Mexico City have imposed bans either temporarily or permanently (Davis, 2008; Despacio and ITDP, 2013; Gu et al., 2017) to address concerns with congestion and/or air quality. In some cities such as Santiago, Chile and Frankfurt, Germany, stringency of bans vary by vehicle ages and types, as older and more polluting vehicles generally face tighter restrictions (Barahona, Gallego and Montero, 2019; Wolff, 2014).

In Latin American cities, command-and-control policies have been more popular than congestion pricing schemes (Mahendra, 2008), despite the fact that price-based mechanisms are viewed as more economically efficient. For instance, Bogota, Caracas, La Paz, Lima, Mexico City and Sao Paulo have all implemented vehicle circulation restrictions for congestion or air quality reasons in the recent past. Buenos Aires and Santiago are the only Latin American cities using market-based approaches to manage demand for driving, but this applies only to specific road facilities. Other attempts at pricing road infrastructure through tolling (e.g. Mexico City) have been used in the context of financing transportation infrastructure and as a cost-recovery tool rather than managing automobile demand.

Public acceptance largely determines the success of the congestion relief policies (Li and Zhao, 2017), and congestion pricing has lower public support than vehicle bans in Latin America (Despacio and ITDP, 2013; Mahendra, 2008). Most researchers agree that market-based approaches are more economically efficient, but raise equity concerns as fees are likely to impact low- and middle-income drivers more heavily than high income drivers (Levinson, 2010). Others suggest that the benefits of congestion pricing for lower-income individuals, as a group, greatly outweigh the increased costs to particular individuals within that group (Schweitzer and Taylor, 2008; Taylor and Norton, 2009). Broadly speaking, studies to date have focused on three aspects related to support of congestion relief policies: the potential cost and benefits and their distribution among users and non-users (King et al., 2007; Levine and Garb, 2002), socio-economic factors (Jakobsson et al., 2000; Schmöcker et al., 2012), and the importance of civic engagement and values, such as environmentalism, in explaining support for policies (Börjesson et al., 2015; Hårsman and Quigley, 2010). The public acceptance towards congestion relief policy schemes may be even more complex in emerging cities than developed cities (Sugiarto et al., 2017).

Despite the high prevalence of driving restrictions in Latin American cities, and the paucity of pricing approaches to manage congestion, it is surprising to see few studies focusing on cities in this region of the world. With increasing interest in pricing as a mechanism to manage congestion and bring local and global environmental and health co-benefits, it is timely to examine the factors that explain support for various congestion relief policies in a largely urban yet unequal region of the world. Accordingly, in this study we examine the individual- and city-level factors associated with the public acceptance of market-based and command-and-control demand-side congestion relief policies in eleven Latin American cities: Buenos Aires, La Paz, Sao Paulo, Fortaleza, Bogota, Quito, Lima, Montevideo, Caracas, Panama City and Mexico City. We contribute to the literature in the following ways. First, this study is the first quantitative and multicity study for Latin America on this topic. Second, while the literature mainly focuses on the market-based congestion relief policies, this study examines both congestion pricing and vehicle restrictions. Third, we examine a broad set of individual-level public health and city-level factors which have not been accounted for in most previous studies. Nevertheless, due to data limitations, this study does not build casual relationships, examine supply-side policies, or further explore the effects of personal beliefs towards inequality; all of them should motivate further research on this topic. In the next section, we review the literature and summarize emerging questions. We then describe the methods and findings, and conclude with a discussion of the findings and implications for policy.

## 2 Market-based and command-and-control congestion relief approaches

Policy makers frequently seek to reduce congestion in order to reduce traffic delay or improve air quality: cities such as Bogota and London introduced such policies initially mainly as a traffic relief policy (Leape, 2006; Mahendra, 2008), while cities such as Santiago and Mexico City started these policies as a tool for mitigating air pollution (Onursal & Gautam, 1997). Such congestion relief policies can either be market-based or command-and-control. In Latin America, command-and-control driving restrictions are more popular than the market-based congestion pricing. Cities including Bogota, La Paz, Lima, Medellin, Mexico City, Quito and Sao Paulo all have various types of driving restrictions (Despacio and ITDP, 2013; Lopez-Ghio et al., 2018). These restrictions are normally based on license plate numbers and are enforced either during the peak-hour only or all-day (Guerra and Millard-Ball, 2017; Zhang et al., 2017). In contrast, in Latin America only Buenos Aires and Santiago have congestion pricing, although they focus only on specific roads or highways (Despacio and ITDP, 2013; Lopez-Ghio et al., 2018) and lack the broader appeal of charging for circulation in a broader area such as London (Givoni, 2011).

The effects of these market-based or command-and-control congestion relief policies are mixed, with some cities seeing a success in reducing congestion and improving air quality (Givoni, 2011). London, for example, saw initial congestion and envionrmental benefits, and has progressively increased the charge to keep up with its ability to manage travel demand, and to decrease vehicular emissions. Other cities such as Beijing and Mexico City have experienced only short-term effects but not long-term ones (Davis, 2008; Gallego et al., 2013; Sun et al., 2014) partly because residents adapt behaviorally and shift to driving at other times or purchase second vehicles (Eskeland & Feyzioglu, 1997; Cantillo et al., 2014; Gu et al., 2017; Moncada & Bocarejo, 2015; Ramos et al., 2017). Real-istically, policy packages combining driving restrictions and road pricing may be most effective in mitigating air pollutions and improving welfares for different social groups, especially when the toll revenues could be used for transit subsidies (Basso, Montero & Sepulveda, 2019; Daganzo, 2000).

Individuals’ support for congestion relief policies is associated with expected or actual personal benefits and costs. From a mobility perspective, travelers facing the most travel delay stand to benefit the most from congestion relief policies. For instance, based on a post-referendum survey data in Stockholm, Hårsman and Quigley (2010) found that a 10% reduction of potential commute time was associated with 2% higher likelihood of supporting a toll system. Yet, car owners were less likely to support such congestion relief policies. According to a post-referendum survey in Edinburgh, only 25% of the car owners voted support for road pricing, while 64% of the non-car owners voted support for the referendum (Gaunt et al., 2007). In addition, such policies will gain higher levels of support if paired with increased accessibility via other modes such as transit (Levine and Garb, 2002). This is intended to mitigate the accessibility losses brought by congestion pricing.

Another personal and social benefit comes from the decreased emissions from reduced driving. In London, the cordon pricing scheme was associated with decreases of NOx and PM_10_ (Beevers & Carslaw, 2005). In addition, Stockholm’s congestion pricing scheme was associated with reductions of children’s respiratory diseases (Simeonova et al., 2018). Stated choice experiments in Jakarta and Nagoya show that being aware of the environmental issues in the city is positively associated with supporting congestion pricing (Sugiarto et al., 2017). Other simulations have identified the environmental benefits of road pricing (Mitchell, 2005; Cipriani et al., 2018). Despite the emerging awareness of these benefits, few studies have connected these health co-benefits with policy acceptance.

Environmentalism and civic engagement are also predictors of congestion relief policy support, as reducing congestion can also bring about social benefits. People with higher levels of environmental awareness are more likely to support congestion relief policies (Giuliano, 1992). In Stockholm, Hårsman and Quigley (2010) found that neighborhoods with 10% higher pro-sustainability party affiliations are 4.6% more likely to support congestion pricing. Similar results were found in a multi-city study in Stockholm, Helsinki and Lyon (Börjesson et al., 2015). Environmental awareness is likely to be confounded with levels of education (Kollmuss and Agyeman, 2002) and income. For instance, Manville and Cummins (2015) found that, in the US, those with college education are more likely to support transit spending. In addition, environmental awareness is theoretically associated with higher levels of civic engagement, including voting turnout (Brehm and Rahn, 1997; Dresner et al., 2014; Knack, 1992).

While market-based congestion relief policies are more economically efficient (Harrington and Morgenstern, 2004), they are not as popular as the command-and-control policies in Latin American cities (Mahendra, 2008). Congestion pricing is likely to have organized opposition from auto owners (King et al., 2007) who also happen to have outsize impact on policy formulation because they have higher income. Other residents might have additional concerns towards congestion pricing and its equity impacts, as travelers with a high value of time and higher income will disproportionately enjoy the travel benefits of the policy (Jakobsson et al., 2000; Levinson, 2010). However, such equity concerns also exist for command-and-control congestion relief policies, since those with higher income can purchase additional vehicles to circumvent the license-plate-based restrictions (Davis, 2008; Gu et al., 2017), although this behavioral adaptation appears to be less salient to the public. Higher income residents may also have increased schedule flexibility to accommodate pricing peaks, while for lower income workers with limited options it makes it more costly to get to work, leading directly to lost wages.

In addition to individual-level factors, city-level attributes are important in understanding public acceptance of congestion pricing and/or driving restrictions. Studies in Australia and Europe both found that public acceptance of road pricing significantly differs by city, controlling for other individual-level characteristics (Börjesson et al., 2015; Zheng et al., 2014). Such city-level differences might be due to different levels of trust towards government (Kim et al., 2013), different policy preferences (Kim et al., 2013), perceived effectiveness of congestion relief policies (Eliasson, 2014), and differences in transportation infrastructure and alternatives to automobile use faced by each city (Lopez-Ghio et al., 2018). Such city-level differences may also impact the relative preference between different approaches. For example, in China, different cities apply different policy tools to manage the number of new license plates issued each year. Beijing, the capital city, uses lottery to assign new license plates, and Shanghai, the economic hub, auctions new car licenses, while other cities such as Guangzhou applies a hybrid system combining the two (Chen and Zhao, 2013; Wang and Zhao, 2017).

In summary, although theoretical discussions and empirical evidence regarding public acceptance towards congestion relief policies is emerging, significant questions remain. First, although there is significant evidence on the public acceptance of congestion pricing, studies comparing driving restrictions and congestion pricing are limited. Many cities are facing questions of how best to manage road traffic, and the choice between market-based and command-and-control policies is prominent. Studies on these topics will not only enrich the literature but also provide empirical evidence for policy makers. Second, most studies focus on the effects of individual-level factors, while cross-city studies are seldomly conducted. Evidence on city-level factors can help reveal differences across cities, and the mechanisms behind them. Third, among the individual-level factors associated with policy support, environmental health considerations deserve attention. Although there are a few studies on the health benefits of congestion relief policies, there is a paucity of studies that connect such benefits with public support. Finally, most existing studies cover one or a limited number of cities in the Global North. Yet, studies in Latin America – another highly urbanized and highly unequal region of the world – are limited. Studies covering multiple cities can be especially helpful to examine difference in city-level factors associated with public support of congestion relief policies. This study aims to fill these gaps by utilizing a unique dataset covering 11 cities in Latin America to examine both individual and city-level factors on public support of market-based and/or command-and-control congestion relief policies.

## 3 Data and methods

### 3.1 Survey data

**Fig. 1:**
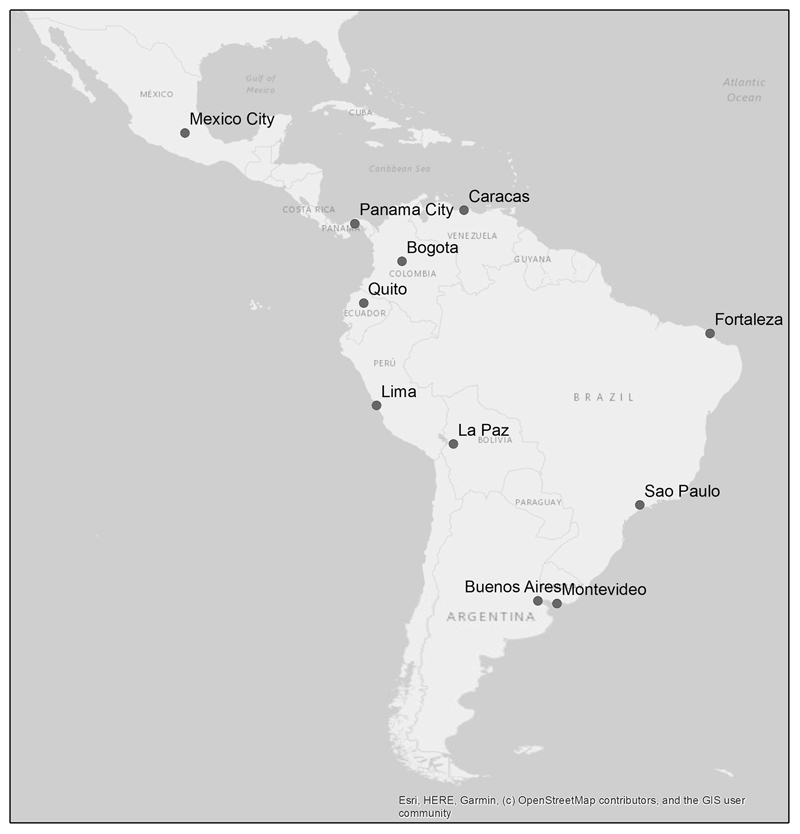
Locations of the 11 cities in the study.

The dataset for this study comes from a 2016 survey conducted by the Development Bank of Latin America *(Corporación Andina de Fomento* or CAF). The survey (“the 2016 CAF survey” hereafter) includes cross-sectional data for 12,905 individuals collected from November 2016 to January 2017 (Development Bank of Latin America, 2017; Wang et al., 2019). This survey covers 11 cities varying in area and population: Buenos Aires (Argentina), Bogotá (Colombia), Caracas (Venezuela), Fortaleza (Brazil), La Paz (Bolivia), Lima (Perú), México City (México), Montevideo (Uruguay), Panamá City (Panamá), Quito (Ecuador) and Sao Paulo (Brazil) (Fig. 1). Sampling was stratified by city. Specifically, the survey adopted a semi-probabilistic multi-stage stratified sampling approach, with random selection of sampling units (e.g. neighborhood) at the sampling point level (e.g. district) and systematic selection of dwellings with random starting points within the sampling unit. More details about the sampling are summarized elsewhere (Development Bank of Latin America, 2017). For each household, only one individual between 20 and 60 years of age was interviewed. For each city, the survey includes between 1000 and 1500 respondents, except for Panamá City which only has 600 respondents. The survey includes 138 questions organized by ten themes: demographic, migration, housing, public and cultural property, employment, education, mobility, safety, civic engagement and health. All data used in the analyses are publicly available.

### 3.2 Statistical modeling

To examine the individual- and city-level factors associated with supporting congestion relief policies, we propose regression models following the equation below:

(1) $$Support_{i}=f\left(\beta_{0}+\beta_{1}\;Personal_{i}+\beta_{2}Civic_{i}+\beta_{3}CityFactor_{i}+\beta_{4}X_{i}\right).$$

In this equation, *Support_i_* is a binary variable indicating whether Respondent *i* supported a specific congestion relief policy or not; here we use three outcome variables: support for congestion pricing (market-based), support for driving restrictions (command-and-control), and support for at least one of the two. *Personal_i_* refers to perceived personal cost and benefits of the proposed congestion relief policies, including whether Respondent *i* used private automobiles, rail/BRT, bus/taxi/informal transit during her/his commute, level of traffic delay, automobile ownership, transit isolation index, and having children with recent respiratory diseases. *Civic_i_* refers to level of civic engagement of Respondent *i*, including voted in the most recent presidential election, and whether her/his household members actively participated in local institutions. *CityFactor_i_* refers to factors of the city that Respondent *i* resides in; it includes either city fixed effects or city-level characteristics (GDP per capita, income inequality, population size, air quality and with driving restrictions in place). *X_i_* refers to control variables, including education level, gender, age, living with partner, having children, employment status and home ownership. Detailed discussions of these variables, including how to construct them, are available in Sections 3.3–3.7 below.

We used logistic regressions since the three outcome variables in this study are all binary. In order to adjust for the potential autocorrelation by city, all standard errors were clustered by city. In other words, we assume that respondents within the same city are correlated with each other, as opposed to assuming all individuals are independent. For instance, it is possible that within each city individuals closer to each other are more correlated than those further apart, and clustering standard errors by city takes this possibility into account. We estimated two sets of models. The first set examines associations between supporting any congestion relief policy (market-based or command-and-control) and personal transportation, children’s health, civic engagement and city-level factors (city fixed effects and city-level characteristics). The second set has the same explanatory variables but separately investigates support for congestion pricing and support for driving restrictions. In each set, we first estimated unadjusted associations between personal transportation and health variables (i.e. *Personal_i_* in Equation (1)); secondly, we added civic engagement/socio-economic variables (i. e. *Civic_i_ and X_i_*) to the first model; finally, we added city-level variables (*CityFactor_i_*) to the second model. We also conducted several sensitivity analyses to ensure the robustness of our findings. All analyses were conducted in Stata 15 (StataCorp., 2017).

### 3.3 Support for congestion pricing and for driving restrictions

We use three outcome variables: a) support for congestion pricing, a market-based congestion relief policy (yes/no), b) support for a command-and-control congestion relief policy, driving restrictions (yes/no), and c) support for at least one of the two (yes/no). These three variables come from two questions of the 2016 CAF Survey. The first question is: *“State your agreement with charging a fee for private vehicles to travel at peak hours if this would help reduce traffic?”* (in Spanish: *“¿ Qué* tan *de acuerdo estaría usted con el cobro de una tarifa a los automoviles particulares por transitar en horas pico/punta si esto ayudara* a *reducir el tránsito?”)* and the second question is *“State your agreement with authorities partially restricting private automobile traffic in the center during peak hours if this would help reduce traffic?”* (in Spanish: *“¿Qué* tan *de acuerdo estaría con que las autoridades restringieran parcialmente el tránsito vehicular de automóviles particulares en el centro durante horas pico si esto ayudara a reducir el tránsito?”*). For each question, the respondent had a 3-point response scale: “agree”, “neutral” or “disagree”. We constructed the dependent variables on supporting the policy as “yes” if the respondent selected “agree,” and “no” if “neutral” or “disagree” were selected. As a robustness check, we also ran ordered probit models for congestion pricing and driving restrictions with the raw 3-point response as dependent variables (results not shown), and significant variables and signs were largely similar to those presented in this paper.

### 3.4 Personal cost and benefits

According to the literature reviewed, perceived personal costs and benefits of the proposed congestion relief policies are associated with individuals’ support for congestion pricing and/or driving restrictions. Among travelers, road public transit users (formal and informal) are likely to benefit as travel times decrease from lower traffic congestion, although demand for those transit modes may increase causing mode-specific congestion. Travelers that use modes that involve exclusive rights of way like rail or bus rapid transit (BRT) may be less likely to support the ban as they might suffer from increased in-mode congestion but may not benefit directly from the travel time savings of decreased traffic. Whether the gains in access and egress times overcome the losses due to station and invehicle congestion is an empirical question. As a result, we include measures of whether travelers use rail/BRT and other transit modes.

We also expect travelers who face the most congestion to be more supportive of congestion relief policies as these individuals stand to benefit the most from it. Specifically, level of congestion for an individual is measured as the share of one’s commuting time that is due to traffic delay. We calculated travel delay time using the difference between self-reported commuting time and the self-reported stated commuting time if there were no traffic. The self-reported congested and uncongested time come from a survey question about the door-to-door travel time during a “normal day” to reach the respondents’ main activity with and without congestion. This main activity was undefined and could be work, school or others. To reduce the potential influence of unrealistic commutes, we truncated those who reported travel times (congested and uncongested) of more than 180 min to 180 min (N = 36). Furthermore, we excluded 36 respondents whose delay-to-uncongested-travel-time ratio was greater than 90%. To validate these self-reported uncongested and congested travel times, we compared them with Google Maps-derived travel time for the actual origins and destinations in a subsample of respondents in Bogota showed moderate agreement (Pearson correlation: 0.62 and 0.70, respectively) (Wang et al., 2019). Furthermore, we compared the Google Maps times with Uber Movement data for Bogota and found almost perfect agreement between both (Pearson correlation: 0.96).

Consistent with our expectation that the private benefits of congestion relief policies increase support, we hypothesize that parents who report whether their child or children that recently have had respiratory diseases will be more likely to support these policies. Hence, we created a dummy variable that equals one if the respondent had a child of 5 years of age or less and who was diagnosed with a respiratory disease in the past two weeks of the survey, and zero otherwise. We also include additional mobility-related variables that we expect are associated with policy support such as auto ownership and transit access. Holding travel delay constant, we expect auto ownership to be negatively associated with support for congestion relief policies. Similarly, access to formal or informal transit is expected to be positively associated with congestion policy support. And conversely, those with limited access to transit are less likely to support the policies as they may be more reliant on private automobiles or other modes. As a result, we followed Wang et al. (2019) and measured transit isolation (no access to formal or informal transit stops within a 10-min walk), which we hypothesize is negatively associated with congestion relief policies.

### 3.5 Civic engagement and socio-economic variables

Prior research suggests that civic engagement is associated with environmental awareness (Brehm and Rahn, 1997; Dresner et al., 2014; Knack, 1992) and hence we hypothesize that those with more civic engagement are more likely to support congestion relief policies. Based on the 2016 CAF survey data, we constructed two variables measuring civic engagement for national- and local-level issues: whether individuals voted in the last presidential election (yes/no) and having a household member frequently participating in a neighborhood association, local council, non-profit, or other local organization to improve safety, lighting, streets, public infrastructure, or the physical appearance of the neighborhood (yes/no).

### 3.6 Other individual-level control variables

We also included seven socio-economic variables from the 2016 CAF as potential controls because they are likely to influence support for the policies and may be correlated with the main explanatory variables described previously: educational attainment (less than high school, high school/some college and college or higher), gender (male/female), age (in years) and squared, living with a partner (yes/no), having children (yes/no), employment status (either full-time or part-time, yes/no), home ownership status (yes/no). Unfortunately, the 2016 CAF has very limited information on personal or household income. Instead, in robustness checks we created a wealth index using the information of durable goods ownership, housing characteristics, and utility access as a proxy of income.

### 3.7 City-level variables

We included ten city-fixed effects variables with Buenos Aires as the reference category. We also included five additional city-level characteristics. The first variable, city-level GDP per capita in 2016 (in 1000 US Dollars), comes from Oxford Economics (2019). We expect this variable to be negatively associated with the support of congestion relief policies, as there are more residents being impacted by such policies in higher-income cities. The second variable, city-level income Gini coefficient, from UN-Habitat (2016), measures the level of income inequality for each city (in 2007, 2009 or 2010). The coefficient ranges from 0 to 1, with a higher number indicating higher income inequality. We expect the Gini coefficient to be negatively associated with the support of congestion pricing and positively associated with support for driving restrictions as the latter are seen as fairer, considering that the CAF survey does not mention whether the tolls will be distributed to transportation infrastructure improvements. However, we acknowledge that it is equally possible that residents living in cities with higher Gini coefficient are more tolerant towards income inequality. Under such hypothesis, city-level Gini coefficient would be positively associated with the support of congestion pricing and negatively associated with support of driving restrictions. The third variable, city-level population in 2016, comes from Oxford Economics (2019). We expect residents of bigger cities are more supportive of congestion relief policies. The fourth variable is the annual median fine particulate matter (PM_2.5_) measured at or around the city center in 2016 (μg/m^3^). For each city, the center was manually identified through Google Map by identifying apparent centers such as city hall, center park and central squares (Google, n.d.). The PM_2.5_ data was retrieved from the Global PM_2.5_ updates (version: V4.GL.02, 0.1° × 0.1° with GWR) as estimated by Van Donkelaar et al. (2016). We expect higher fine particle concentrations to be positively associated with support for both market-based and command-and-control congestion relief policies. The fifth variable is a dummy variable indicating whether the city currently has or has had driving restrictions (yes/no) due to congestion or air quality concerns. This variable is constructed based on Despacio and ITDP (2013) and Mahendra (2008) and updated based on newspaper reports. We expect places that have had congestion relief policies to be supportive of those policies, perhaps through a process of adaptation and normalization.

## 4 Results

### 4.1 Descriptive statistics

The final sample of study includes 8178 individuals with complete information on congestion relief policy support, personal transportation factors, civic engagement information, and socio-economic characteristics. A total of 4727 individuals (out of the total 12,905 in the original CAF 2016 survey) were excluded from the analysis because 636 did not have information on congestion relief policy support, 3869 had missing data on personal or transportation factors, 86 had missing data on civic engagement, and 136 were missing socio-economic characteristics. The share of survey respondents supporting congestion relief policies is slightly higher for those in the study sample than those excluded. However, the share of the using mass transit to commute, levels of traffic delay during commute and the share of children (5 or younger) having respiratory diseases are comparable between these two groups (Table A1 in Appendix).

**Fig. 2:**
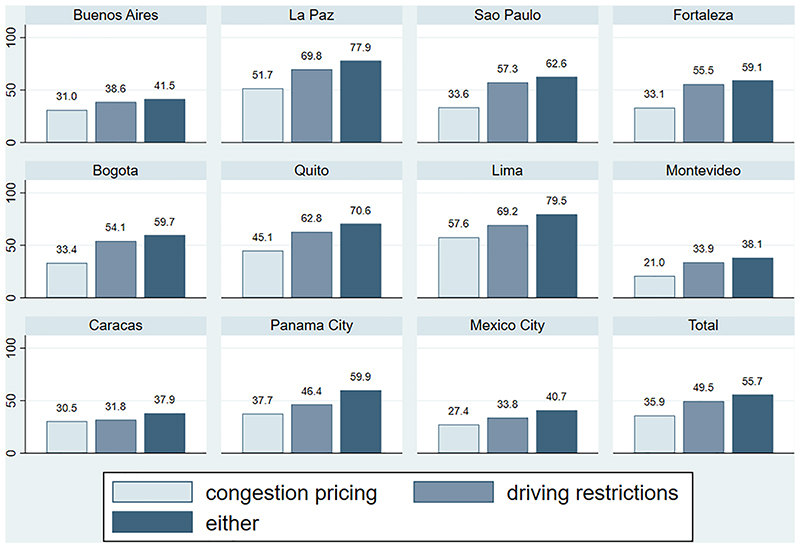
Percent (%) supporting congestion pricing, driving restrictions, or either; stratified by city (N = 8178).

Fig. 2 shows the share of support for congestion pricing, driving restrictions, or either in the study sample. It shows considerable variation across cities, with support for either as high as 77.9% in La Paz and as low as 37.9% in Caracas. Despite such differences across cities, driving restrictions (49.5% for the full sample) are consistently more popular than congestion pricing (35.9% for the full sample). This level of support for congestion pricing is comparable with what has been reported for Helsinki (35%) and Lyon (32%), but lower than Stockholm (68%) (Börjesson et al., 2015). The largest gap between congestion pricing and driving restrictions support is in Sao Paulo, where there is 23.7 percentage points of additional support for driving restrictions.

For transportation factors, descriptive statistics suggest that formal and informal public transportation and taxis were the most common modes of transportation for all cities except Bogota, where BRT was most common. On average, 14% of the respondents used a private automobile during their commute (Table 1). Overall, 33% of the respondents owned automobiles, with a high of 50% in Sao Paulo and a low of 19% in Lima. While 14% of respondents did not have access to transit within a 10-minute walk from home, for some cities like Quito and La Paz this was as high as 23%, and for others like Fortaleza (3%) and Montevideo (4%) it was low.

Among the respondents, 7% of them had children up to 5 years old suffering from respiratory diseases in the past two weeks, although La Paz (13%) and Lima (11%) respondents reported the highest percentage and Montevideo and Panama City the lowest (4%.) As to civic engagement, 83% of respondents voted in the past presidential election, with La Paz (96%) and Lima (95%) having the highest percentages and Bogotá (59%) and Panama City (61%) the lowest. Around 17% of respondents had household members engaged locally with institutions to improve neighborhood conditions, with La Paz (39%) and Mexico City (33%) with the highest percentage and Montevideo (5%) and Fortaleza (3%) with the lowest percentage.

For socio-economic characteristics, only 11% of respondents had a college degree, average age was 37.1 years, and 51% of respondents were female. At the city level, there is considerable heterogeneity in GDP per capita and income distribution, with the GDP per capita ranging from 3270 US Dollars in La Paz to 19,770 US Dollars in Fortaleza, and the Gini coefficient ranging from 0.38 in Caracas to 0.60 in Fortaleza. City population ranged from 440 thousand for Panama City to 21.3 million for Sao Paulo. The city with the highest city-center annual median PM_2.5_ was Lima (32.2), and the lowest was Caracas (4.8). Six cities (Buenos Aires, Bogota, Lima, Mexico City, Montevideo, Sao Paulo), including the largest ones in terms of population, exceed the World Health Organization air quality guidelines for PM2.5 of 10 μg/m^3^ mean per year. Finally, five of the eleven cities had vehicle circulation bans at the time of survey: La Paz, Sao Paulo, Bogota, Quito, and Mexico City.

### 4.2 Support for congestion relief policies: transportation, health and city-specific factors

The outputs of the regressions for the support for congestion relief policies are shown as Models 1–4 in Table 2. The fully-adjusted models (Models 3–4) are able to correctly classify 62%-65% of the observed choices; also, the AUC of Models 3-4’s ROC curves are 0.67–0.69 (ROC curves not shown), showing moderate levels of goodness-of-fit.

As expected, some individual characteristics that represent personal cost and benefits of congestion relief policies significantly predict the support for those policies. Specifically, private automobile users are less likely to support congestion relief policies (Table 2, Model 1). By contrast, those spending a larger proportion of commute time stuck in traffic are more likely to support congestion relief policies. Neither use of any transit service, transit isolation, nor auto ownership are statistically significant, although car ownership becomes significant as other covariates are included in the model (Models 3 and 4). Whether respondents had any children (up to five years old) suffering from respiratory diseases in the past two weeks of survey is positively associated with support for congestion relief policies (Model 2). The associations described hold even after controlling for socio-demographic characteristics of respondents. Contrary to expectations, respondents’ civic engagement as measured is not associated with support for the policies (Model 3).

Based on Model 3 which includes city fixed effects, holding continuous covariates at their mean values and non-continuous covariates at their modes, the average marginal effects suggest that car users for the commute are 10.8% less likely to support congestion relief policies than non-car commuters, while car owners are 3.7% less likely to support the policies than non-car owners. In addition, every 10% higher in traffic delay – commute time ratio is associated with 0.6% higher likelihood to support the policies, regardless of the travel mode used. We examined whether this association between travel delay and congestion policy support was moderated by the car use but did not find an association (results not shown). Finally, having a child with respiratory disease in the past two weeks was associated with 5.9% more likely to support congestion relief policies.

City-level factors are also associated with level of support for congestion relief policies. As shown in Models 3 and 4 (Table 2), between-city differences in congestion relief policy support tend to be larger in magnitude than those across different personal characteristics. After adjusting for personal socio-economic characteristics, travel patterns, children respiratory diseases and civic engagement, policy support is highest in Lima and La Paz, and lowest in Caracas and Montevideo. Model 3 in Table 2 indicates that, holding other factors at their means or modes, a resident in Lima was 35.1% and a resident in La Paz was 33.4% more likely to support congestion relief policy than a Buenos Aires counterpart. Similarly, a resident in Caracas was 4.4% and a resident in Montevideo was 3.9% less likely to support congestion relief than that in Buenos Aires.

Model 4 in Table 2 provides a more nuanced understanding of city-specific characteristics associated with the support of congestion relief policies. Income inequality, rather than per capita GDP, is significantly associated with individual-level support for the policies; in addition, higher levels of fine particulate matter at the city center and prior exposure to driving restrictions are both associated with higher likelihood of individual support for the policies. Specifically, a 0.1 increase in the city-level Gini coefficient is associated with 5.6% higher likelihood of support, while holding other factors at their means or modes. Similarly, a 1 μg/m^3^ increase in PM 2.5 concentration is associated with 1.5% higher likelihood of support; and living in a city that already has driving restrictions is associated with a 14.2% higher likelihood of support.

It is notable that among the individual-level socio-demographic characteristics included in the models, neither level of education nor employment are associated with policy support, with or without adjusting for city-level variables (results not shown). As a sensitivity analysis, we re-ran the models without personal transportation/health or civic engagement variables, and the coefficients of education and employment remain insignificant (results not shown). In addition, females are consistently less supportive of either policy than males. It appears that more proximal, behavioral choices like commuting patterns, or city-level environmental and income distributional characteristics are better able to explain support for congestion relief policies than variables reflecting general socio-economic status.

### 4.3 Congestion pricing vs. Driving restrictions? Effects of city-level factors

Thus far we have treated congestion pricing and driving restrictions together, even though they are implemented differently and as a result are likely to have disparate effects on subpopulations and different levels of support. We now consider support for each policy separately in order to identify salient differences. Table 3 shows two models (Models 5–6) for support for congestion pricing and two models (Models 7–8) for driving restrictions, controlling for personal transportation factors, children’s respiratory health, civic engagement variables, socio-economic characteristics, and city-level factors (either city fixed effects or city-specific factors). These four models are able to correctly classify 62%-66% of the observed choices (Table 3); also, the AUC of the ROC curves of the models are 0.62–0.67 (ROC curves not shown), showing moderate levels of goodness-of-fit. Differences in predictors of support across policies are largely due to factors at the city level, rather than the individual level. The only consistent difference in personal factors is that commute delay is positively associated with support for driving restrictions whereas it is not associated with congestion pricing. Wald test of differences of the coefficients based on seemingly unrelated probit regressions shows that the effects of personal transportation and health factors do not differ by policy, while those of all ten city-fixed effects are significantly different between the two policies. Among the eleven cities, Caracas and Mexico City are the only cities where support for circulation bans are lower than for pricing.

For city-level characteristics, as Models 6 and 8 indicates, GDP per capita is negatively associated with supporting congestion pricing but not driving restrictions, while income inequality is positively associated with supporting driving restrictions but not congestion pricing. Holding continuous covariates at their means and binary variables at their modes, Model 6 indicates that a 1000 USD increase of GDP per capita is associated with 0.8% lower likelihood of supporting congestion pricing; and Model 8 indicates that an 0.1 increase of the city-level Gini coefficient is associated with a 7.2% higher likelihood of supporting driving restrictions. Other city-level characteristics are similarly associated with supporting congestion pricing and driving restrictions, with having a circulation restriction and higher levels of fine particulate matter concentration positively associated with support for both policies. However, note that the association between having driving restrictions and supporting congestion pricing is significant at a 10% level (p = 0.09). City population size is not associated with either policy.

**Fig. 3:**
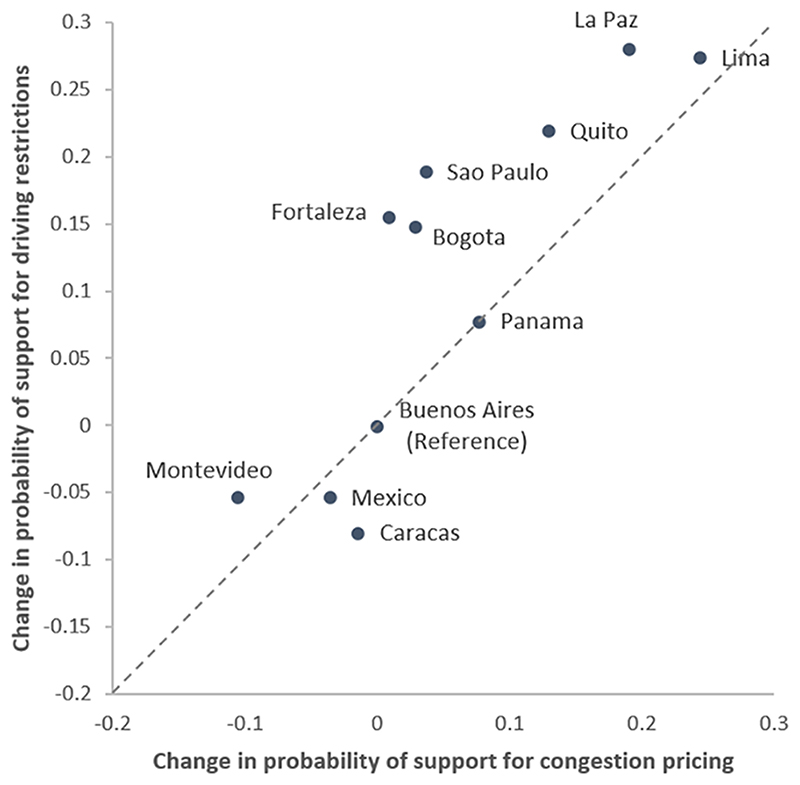
Change in probability of policy support. Note: Estimated average marginal effects are relative to a Buenos Aires resident. Estimations are based on Model 5 and Model 7 in Table 3 with continuous covariates at their means and categorical covariates at their modes.

To visualize differences among city residents in support for congestion pricing and driving restrictions identified in Models 5 and 7 (Table 3), we calculated the average marginal effects of the city coefficients and plotted them in Fig. 3. Marginal effects are calculated with other covariates at the means (continuous) or modes (categorical). In Fig. 3, Buenos Aires (the reference category) is centered at (0,0). The x-axis shows whether other cities have higher or lower propensity to support congestion pricing relative to Buenos Aires. The y-axis shows whether the same cities have a higher or lower propensity to support command-and-control congestion policies. For instance, on average a Lima resident is 24.4% more likely to support congestion pricing and 27.4% more likely to support driving restrictions than her/his counterpart in Buenos Aires; also, one living in Montevideo is 10.6% less likely to support congestion pricing and 5.4% less likely to support driving restrictions than her/his counterpart in Buenos Aires, all else held equal. Fig. 3 also confirms that the stronger preference towards command-and-control policies remains after adjusting for all other covariates. These cities include, for instance, Sao Paulo (18.9% higher for driving restrictions, 3.6% higher for congestion pricing, both relative to Buenos Aires), Fortaleza (15.6% higher for driving restrictions, 0.8% higher for congestion pricing) and Bogota (14.8% higher for driving restrictions, 2.9% higher for congestion pricing). In contrast, residents in Caracas (8.0% lower for driving restrictions, 1.5% lower for congestion pricing) and Mexico City (5.3% lower for driving restrictions, 3.6% lower for congestion pricing) prefer pricing-based policies.

**Fig. 4:**
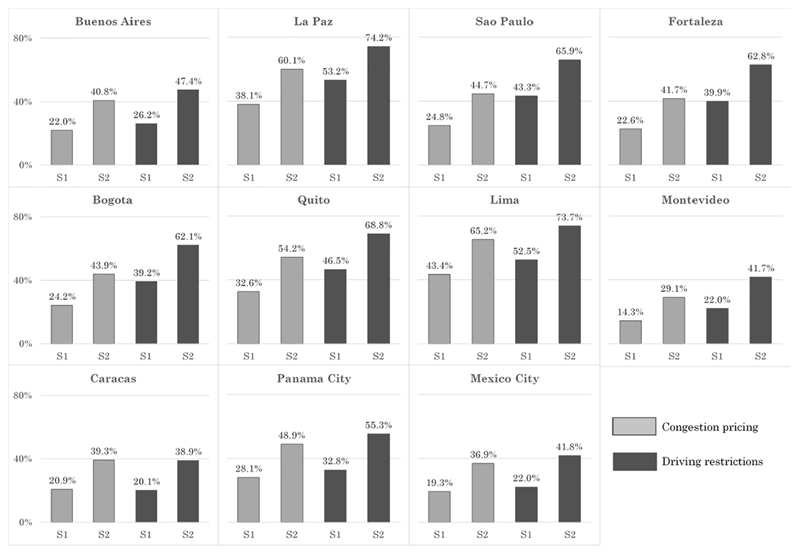
Predicted probability of policy support by city. Note: Estimated based on Models 5 and 7 and in Table 3. S1 and S2 refer to different scenarios. S1: own automobiles, commute by private auto, 33% of commute time stuck in traffic (sample median), does not have child 5 years of age or less suffering from respiratory diseases. S2: does not own automobiles, does not commute by auto, 56% of commute time stuck in traffic (90th percentile), has child 5 years of age or less suffering from respiratory diseases. Other covariates equal to means (continuous) or modes (categorical).

To further illustrate differences across cities we estimated the predicted probability of supporting each policy for a hypothetical resident in each city under two different scenarios (Fig. 4) using the coefficients from Models 5 and 7 in Table 3. In the first scenario (S1), the resident owns an automobile and commutes by private automobile, is delayed by congestion by 33% more than free flow time (median delay in the sample), and does not have a child 5 year of age or younger suffering from respiratory diseases. In the second scenario (S2), the resident does not own an automobile, does not commute by private automobile, is delayed by congestion by 56% more than free flow time (90th percentile in the sample) and has a child 5 year of age or less suffering from respiratory diseases. All other covariates are held at their means (continuous) or modes (categorical). As expected the predicted probabilities vary significantly across cities and scenarios (Fig. 4). In every case there is more support for driving restrictions than for congestion pricing. Support for congestion pricing is lowest in Mexico City (scenario 1, 17.8% of respondents) and highest in Lima (scenario 2, 65.2% of respondents). Support increases from the first scenario to the second, with the lowest increase of support of 14.7 percentage points (Montevideo) and the highest increase of 22.0 percentage points (La Paz). Support for driving restrictions is lowest in Caracas (scenario 1, 20.9%) and highest in La Paz (scenario 2, 74.2%). Support increases from the first scenario to the second one, with the lowest increase of support of 18.8 percentage points in Caracas and the highest increase of 22.8 percentage points for Fortaleza and Bogota.

### 4.4 Robustness checks

We conducted several sensitivity analyses to test the robustness of our findings. For the models with city-level characteristics, we also estimated multilevel random-intercept models (with robust standard errors), and the magnitudes and significances of the coefficients remain similar. We also ran models with survey weights and strata, and although the signs of the coefficients remain consistent, the effect sizes slightly changed; however, we were not able to estimate standard errors because of the single-observation strata in the survey sample. Nevertheless, as argued by Solon et al. (2015), since our intent was to examine the variables associated with policy support, rather than make city population-level inferences, we used the unweighted data. For Models 5–8, we have also estimated ordered probit models for the raw opinions (in 3-point scales: oppose, neutral, support) towards congestion pricing and driving restrictions. Significant variables and signs were largely similar; except for Gini coefficient in Model 6 which became significant in the ordered models, and the personal health considerations whose standard error increased but remained significant except for Model 7. In both cases, the signs of the coefficients remain unchanged. In addition, we estimated variance inflation factors (VIF) for the fully-adjusted model and did not find concerns with multicollinearity. Finally, we created a wealth index as a proxy of household income, and re-ran the regression models with this index as a robustness check. Following Vaz et al. (2019), the wealth index is created based on the availability of consumer durable goods, housing characteristics, and access to public utilities. This index is only available for a subset of the study sample without missing data. For the models including this wealth index, the signs and significances of nearly all coefficients remain unchanged. The only exception is that the coefficients for auto ownership become insignificant; this is likely due to the fact that automobile ownership is used in creating the wealth index.

## 5 Discussion

Using 2016 survey data covering 11 Latin American cities, we found that support for congestion pricing and for driving restrictions is associated with both individual- and city-level factors. *Ceteris paribus*, individuals likely to gain the most through reduced traffic delay and personal health benefits were more likely to support those policies, while individuals bearing higher costs of the policies were less likely to support them. In addition, acceptance of congestion pricing and driving restrictions varies significantly by city; and living in a city with higher concentrations of fine particulate matter and having driving restrictions in place is associated with a higher likelihood to support these two policies. Importantly, living in a city with higher GDP per capita is associated with lower likelihood of supporting congestion pricing but not driving restrictions, while living in a city with higher income inequality is associated with higher likelihood of supporting driving restrictions but not congestion pricing.

That respondents facing more severe congestion during the commute and those having children suffering from respiratory diseases are more supportive of congestion relief policies accords well with the argument that these two policies are tools for both traffic management and air pollution mitigation (Givoni, 2011; Mahendra, 2008; Sun et al., 2014). The findings connecting higher congestion during the commute, automobile ownership and commuting using cars with varying support for congestion relief policies is in line with the studies on public acceptance in congestion pricing in Stockholm (Hårsman and Quigley, 2010), Edinburgh (Gaunt et al., 2007) and a multi-city study for Helsinki, Lyon and Stockholm (Börjesson et al., 2015). The interaction term between the travel delay and city having driving restrictions are negative and statistically significant in models for supporting congestion pricing, driving restrictions and either; in particular, the traffic delay-congestion pricing support association is insignificant in cities having driving restrictions, but still significant in cities have no driving restrictions. This is likely to due to the fact that in cities already having driving restrictions, residents still facing severe congestions have lower level of confidence in the effectiveness of congestion relief policies.

Our findings regarding environmental health and support for congestion relief policies highlight the importance of connecting these concepts in the public discourse. To our knowledge, there have been no other studies examining the associations between children’s respiratory health and congestion relief policy support. Nevertheless, these findings are consistent with research showing that the adoption of congestion pricing schemes was associated with lower incidence of children’s respiratory diseases in and Stockholm (Simeonova et al., 2018). Similarly, the finding that higher PM_2.5_ concentrations are associated with higher likelihood of supporting congestion pricing and driving restrictions underscores environmental importance of these two policies. These environmental benefits are also likely to have positive distributional effects (Manville & Goldman, 2018), as non-drivers and drivers alike will benefit from the improved air quality.

We found that city-level factors, rather than the individual-level ones, seem particularly important in explaining support for congestion relief policies – especially for the preference between congestion pricing and driving restrictions. The magnitudes (and marginal effects) of city fixed effects or city-level variables are larger than individual-level variables. We re-ran Models 3, 5 and 7 using two sub-samples by grouping cities with similar city-fixed effects in Model 3 (Table 2). One group includes cities with zero or negative city fixed effects: Buenos Aires, Montevideo, Caracas, Panama City, Mexico City; the other group includes six cities with positive city fixed effects: La Paz, Sao Paulo, Fortaleza, Bogota, Quito and Lima. The aforementioned patterns still hold in these sub-sample regressions (results not shown).

Such findings imply that on top of personal costs and benefits, people living in the same city tend to have to have systematic views regarding their support for specific congestion relief policies. They also suggest that local contexts are critical in determining the political viability of such policies. First, the cities studied have different levels of economic development, automobile ownership, transit service quality, and face unique city-specific challenges (Lopez-Ghio et al., 2018). Such city-level differences are associated with distinct policy choices.

Second, the cities have different institutional settings, political culture and trust towards government, which can shape the relative preference towards these two policies (Kim et al., 2013; Wan et al., 2017). Besides self-interest, personal values towards egalitarianism and/or efficiency are also associated with voters’ policy preferences (Manville and Levine, 2018). People in different cities are likely to have collective identities towards fairness vs. efficiency, and such city-level collective preferences are caught in city fixed effects or city-specific factors. Institutional and political differences across cities are likely to impact not only the acceptability of the policies but also the potential welfare impacts. If suitable substitutes are not available, older, less efficient vehicles will remain in the market for longer, and will be used at other times, with counterproductive results. Thus, even though driving restrictions may be less economically efficient than congestion pricing, they garner more public support. The finding that city-level income inequality correlates with support for driving restrictions but not with support for congestion pricing also underscores the importance of local institutions. This finding suggests that residents from cities with higher Gini coefficients may be concerned about income inequality. This hypothesis could be further tested if data on individual’ s values towards redistributive policies are available for future research. This finding also suggests that income inequality may be a critical population attribute to monitor as congestion relief policies are crafted and proposed. Even though equity issues around congestion pricing can be managed through the use of funds raised (Levinson, 2010), this needs to be communicated effectively with the broader public to gain acceptance. In China, Guangzhou’s experience with the allocation of vehicle registration permits, learning from Beijing and Shanghai, points to the importance of incorporating notions of fairness and equity, not only efficiency, in the management of urban space (Li and Zhao, 2017).

Third, there seem to be “status quo biases” in people’s preferences towards different congestion relief policies (Börjesson and Kristoffersson, 2018; Eliasson, 2014). That is to say, residents in a city without congestion relief policies might over- or under-estimate the effectiveness of such policies. This argument can be inferred by the significant associations between driving restrictions existence and congestion relief policy support in our study. It points to the usefulness of temporary demonstration or pilot projects as a successful way to implement policy incrementally, especially considered that those from the cities with higher level of GDP per capita were less likely to support congestion pricing. Stockholm capitalized on this approach in implemented its cordon pricing scheme.

People’s preferences between price-based and command-and-control congestion relief policies are likely to be the combination of personal interests and intrinsic values (Manville and Levine, 2018). The intrinsic values could either be observed individually or collectively as the models in this study suggest. Theoretically, driving restrictions are regarded as more fair and congestion pricing is regarded as more efficient. However, a person holding more egalitarian values would think twice in supporting driving restrictions if she/he have to drive to work every day. In this case, personal interests and beliefs goes against each other and the person’ s final opinion is likely to the combination of the two. Transportation planners and policy makers should consider such complex dynamics when designing and promote specific policies. Effective congestion relief policies may incorporate both price-based and command- and-control elements, as well as both demand-side and supply-side interventions (Mahendra et al., 2011). For instance, when policies such as congestion pricing or driving restrictions are implemented, increasing transit coverage and service frequency provides an alternative to impacted drivers and can hence ensure the policies’ equity and political acceptability (Mahendra, 2008). Basso, Montero and Sepulveda (2019) proposed a congestion relief policy package by imposing driving restrictions but allowing newer and cleaner vehicles to pay tolls to access the road for “banned” days, and the toll revenue will be used to reduce the transit fares. In their welfare analysis for Santiago, Basso et al. (2019) proved that such policy packages could make individuals of all income groups, with and without cars, better off.

We did not find significant associations between congestion policy support and transit coverage, transit use, or BRT fares (subsample analysis as BRT fare is only available for 10 cities, results not shown). One explanation is that this effect is associated with variations in car ownership and use. Another explanation is that the effect on transit users is ambiguous as these policies may bring benefits and costs to them. This might be particularly important because many Latin American transit systems are well-utilized and crowded (Lopez-Ghio et al., 2018). This finding suggests that travel benefits to transit users need to be magnified to gain the support of this important segment of the population. Distinguishing between transit use and transit service that uses exclusive rights of way (such as BRT and rail) from regular bus service may be important in clarifying these relationships. Similarly, we did not find civic engagement a significant predictor of supporting congestion pricing or driving restrictions. This association have been posited by theory but have yet been identified in empirical studies (Brehm and Rahn, 1997; Dresner et al., 2014; Knack, 1992), although there are studies showing that concerns over environmental issues is associated with higher support of congestion pricing (Börjesson et al., 2015; Hårsman and Quigley, 2010). There is empirical evidence that people’s underlying values towards fairness or efficiency are associated with their support to congestion relief policies in Southern California (Manville and Levine, 2018). Hence, it is possible that variables directly measuring people’s values towards social equity could be a more significant predictor than civic engagement. Our study does not have such variables, and future studies should consider collecting and testing them. Furthermore, we did not find social status variables such as education or employment are associated with policy supports. Our finding that females are less likely than males to support both congestion relief policies are different from the findings by Hårsman and Quigley (2010) that males are less likely to support congestion pricing than females.

The findings of this study shed light on multiple strategies for policy makers to promote the public acceptance of congestion pricing and driving restrictions. First, effective congestion mitigation should move beyond socio-demographic characteristics and focus on both the benefits and costs to individuals and society at large. Although it has been suggested that residents are likely to be more sensitive to the costs than benefits of congestion pricing (King et al., 2007), in this case the variables with the most predictive power (based on coefficients from z-scores for continuous variables and marginal effects for dummy variables, results not shown) are those related to having a previous circulation ban in place and the promise of the ban to improve air quality. Thus, an effective policy should highlight the societal benefits of the intervention, while bundling costs (e.g. monetary cost of auto owners or requiring to shift modes) with targeted benefits aimed at mitigating the impacts (e.g. less crowded, more frequent bus service). Use of the revenues raised thus continues to be a critical factor for determining support of pricing strategies. That travel delay, irrespective of travel mode used, was an important predictor of driving restrictions underscores the benefits of circulation bans across travel modes, potentially broadening the appeal of the policy.

Second, the public health benefits of congestion relief deserve more attention not only to motivate congestion relief policy adoption, but to justify it to the public at large. By improving air quality, such policies are able to bring important health benefits to the population. Especially, considering that residents in cities with higher level of economic performance (measured by GDP per capita) were less likely to support congestion pricing policies, informing them about the public health benefits will be particularly helpful in promoting policies to mitigate traffic.

Finally, the findings regarding income inequality and differences in policy support suggest that the equity concerns surrounding congestion relief policies go beyond first or second-order impacts. To date, most transportation policy literature has focused on the distributional impacts of congestion relief policies on low income drivers (Anas & Lindsey, 2011; Taylor and Kalauskas, 2010). Importantly, recent research has expanded the view to consider how changes in air quality due to congestion relief policies disproportionately benefit low income households (Manville and Goldman, 2018). Our results take concerns about equity in congestion relief policies one step further, highlighting the importance of policy design for public support. As income inequality increased, public support for driving restrictions increased but did not change for congestion pricing. Chen and Zhao (2013) examined vehicle license auctions in Shanghai and noted that although individuals in their sample perceived the policy as effective, they were unsupportive partly due to equity concerns. Among the recommendations provided was to consider driving restrictions in congested locations; our results are consistent with their findings and recommendations. Effective and publicly acceptable policies are context-specific and reflective of the city’s characteristics and challenges.

This study has the following limitations, which should motivate further research. First, although the two questions (driving restriction vs. congestion pricing) focused on peak hour concerns, their geographic coverage was not directly comparable. The question on driving restrictions was more narrowly focused on accessing the city center, whereas the congestion pricing question involved any automobile use. This may help explain why traffic delay did not explain support for congestion pricing. Second, due to the cross-sectional nature of the dataset, we can only interpret the relationships examined as associations. Longitudinal data covering multiple cities could help to identify, for example, whether changes in auto ownership lead to changes in support for particular relief policies. Third, it may be helpful to understand the mechanisms through which income inequality, as measured by the Gini coefficient, seems to favor driving restrictions over pricing. Is it this the result of responses by low income residents, or is it a generalized response among the population? Admittedly, we did not have reliable income information or occupation to address this question. Nevertheless, our models did adjust for educational attainment and the further including a wealth index in the robustness check did not change the conclusions. Fourth, this study only focuses on two demand-side congestion relief policies (congestion pricing and driving restrictions) due to data availability. The survey did not include data for supply-side policies such as (additional) transit subsidies, other demand-side policies such as parking pricing or vehicle distance-based surcharge, and more complex policy packages combining various policy instruments. Future studies should consider conducting stated preference surveys for different policy packages incorporating supply-side and demand-side policy elements. Furthermore, we were not able to include variables on price or quality of each city’s transit system due to a lack of data covering all 11 cities, future studies should consider collecting relevant data. Fifth, the survey provided no information on the potential uses of the revenue. This may have impacted the support for congestion pricing, where the use of revenues is critical in determining support.

## 6 Conclusions

Using self-reported survey data from 11 Latin American cities, we found that the support for congestion relief policies was associated with perceived personal mobility costs and benefits (automobile ownership and use, travel delay due to congestion), personal health considerations (children’s respiratory health), and city-level factors (air quality, income inequality, GDP per capita and currently having driving restrictions). However, neither public transportation uses nor civic participation were associated with support for either policy. When comparing price-based and command-and-control congestion relief policies, support for these two vary by city-level characteristics but not by personal-level ones. Holding other variables constant, residents in Lima and La Paz exhibited the highest support for congestion pricing and driving restrictions, respectively; while residents in Montevideo and Caracas have the lowest support of congestion pricing and driving restrictions, respectively. Residents of cities with higher concentration of fine particles around the city center area are more likely to support pricing and driving restrictions. In addition, residents of cities with higher level of income inequality were more likely to support driving restrictions; however, city-level income inequality was not associated with support of congestion pricing. In contrast, residents of cities with higher GDP per capita is less likely to support congestion pricing, but city-level GDP per capita was not associated with support of driving restrictions. Admittedly, due to limitations of the data, this study does not establish causal relationships between factors of interest and congestion relief policy support; the study only focuses on demand-side congestion relief policies; we were also unable to examine the effects of personal values towards inequality on policy support. If data permits, future studies should focus on these questions building on the current findings and deepening connections to the relevant literature.

To guide implementation of these policies, we identify four important lessons for policy-makers. The first is the importance of addressing costs for those affected, as this is likely to increase their support, whereas those that benefit more already appear more supportive of such policies. Public transit investments seem particularly important given that they will improve support of transit users concerned with a surge in transit demand and in-vehicle congestion, and auto users confronted with the reality of higher out of pocket costs to drive. For congestion pricing, informing the residents on how the fund will be used will be quite helpful. The second lesson is to use the personal and social air quality and associated health benefits as a way to highlight the broader benefits of congestion relief policies. These benefits are widely distributed among the entire population and are likely to be well-received, especially considered that congestion pricing is less popular in cities with better economic performance. The third lesson is to consider how city-level inequality may impact perceptions of policy fairness and desirability. At high levels of income inequality, market-based mechanisms like congestion pricing may encounter less public support. And the fourth lesson is to consider the use of demonstration or pilot projects to test congestion relief policies. This requires a smooth implementation, which, if attained, is likely to underscore the benefits of the policy and garner increased support.

## Supplementary Material

Appendix A

## Figures and Tables

**Table 1:** Characteristics of the study sample, stratified by cities (N = 8178).

Variable	Mean or proportion											
	All	BA	LAP	SP	FOR	BOG	QUI	LIM	MVD	CCS	PAC	MEX
***Personal transportation factors***												
Used private automobiles in commute^±^	0.14	0.13	0.08	0.17	0.10	0.16	0.12	0.06	0.15	0.15	0.24	0.19
Used rail or BRT in commute^±^	0.19	0.11	0.05	0.17	0.01	0.43	0.20	0.11	0.00	0.35	0.40	0.33
Used bus/taxi/informal transit in commute^±^	0.50	0.47	0.78	0.43	0.51	0.24	0.66	0.56	0.51	0.53	0.47	0.52
Share of traffic delay in commute time	0.28	0.16	0.33	0.25	0.26	0.33	0.34	0.29	0.24	0.25	0.40	0.30
Own automobiles	0.33	0.35	0.32	0.50	0.21	0.30	0.29	0.19	0.37	0.25	0.46	0.46
No transit access within 10 min walk	0.14	0.07	0.23	0.12	0.03	0.22	0.23	0.12	0.04	0.20	0.10	0.11
***Personal health consideration***												
Children (5 or younger) has respiratory diseases in past two weeks	0.07	0.05	0.13	0.07	0.05	0.04	0.07	0.11	0.04	0.06	0.04	0.06
***Civic engagement***												
Voted in last presidential election	0.83	0.86	0.96	0.82	0.89	0.59	0.92	0.95	0.92	0.86	0.61	0.73
Household member actively participates in local institutions to improve neighborhood	0.17	0.05	0.39	0.08	0.03	0.20	0.25	0.20	0.05	0.20	0.15	0.33
***Socio-economic characteristics***												
Education (%)												
*Less than high school*	0.39	0.55	0.21	0.36	0.58	0.36	0.58	0.18	0.56	0.27	0.27	0.42
*High school/some college*	0.50	0.42	0.59	0.54	0.39	0.49	0.37	0.72	0.35	0.62	0.52	0.47
*College or higher*	0.11	0.03	0.20	0.10	0.03	0.16	0.05	0.10	0.09	0.11	0.22	0.12
Female	0.51	0.48	0.48	0.49	0.52	0.58	0.49	0.50	0.55	0.49	0.47	0.48
Age (years)	37.1	37.3	35.1	37.0	37.9	38.3	36.4	35.9	37.8	37.2	37.4	37.4
Living with partner	0.59	0.63	0.58	0.55	0.60	0.57	0.62	0.59	0.57	0.61	0.51	0.67
Household with children	0.73	0.77	0.68	0.71	0.78	0.72	0.75	0.72	0.71	0.76	0.69	0.70
Employed	0.67	0.71	0.70	0.66	0.66	0.65	0.56	0.63	0.66	0.68	0.69	0.66
Homeowner	0.67	0.69	0.57	0.60	0.67	0.64	0.54	0.68	0.58	0.84	0.74	0.77
***City-specific characteristics***												
GDP per capita (in 1000 USD)	13.28	17.30	3.36	19.23	8.53	12.86	9.94	9.43	17.91	13.14	18.60	14.55
Income Gini coefficient	0.49	0.51	0.57	0.55	0.60	0.54	0.51	0.40	0.43	0.38	0.46	0.49
City population (million)	8.28	14.55	1.94	21.32	3.91	8.20	1.84	11.10	1.36	3.30	0.44	20.25
Annual median PM_2.5_ around center (μg/m^3^)	13.03	18.50	6.60	16.20	8.90	12.40	8.90	32.20	12.50	4.80	6.30	12.50
City has driving restrictions (1 = yes)	0.44	0	1	1	0	1	1	0	0	0	0	1
***N***	8178	1022	751	815	607	1055	506	717	824	1040	377	464

Note: Acronyms for cities: BA – Buenos Aires, LAP – La Paz, SP – Sao Paulo, FOR – Fortaleza, BOG – Bogotá, QUI – Quito, LIM – Lima, MVD – Montevideo, CCS – Caracas, PAC – Panamá City, MEX – México City.

± Percentages do not add to 100 because categories are not mutually exclusive. Respondents selected all modes used.

**Table 2:** Logistic regressions of support for congestion relief polices (pricing or driving restrictions; N = 8178).

	(1) Transport & health factors only	(2) (1) + civic engagement/socioeconomics	(3) (2) + city fixed effects	(4) (2) + city-specific factors
***Personal transportation factors***				
Used private automobiles in commute	–0.559***	–0.598***	– 0.454***	–0.462***
	[0.152]	[0.140]	[0.111]	[0.108]
Used rail or BRT in commute	– 0.103	– 0.105	0.028	0.048
	[0.175]	[0.156]	[0.092]	[0.096]
Used bus/taxi/informal transit in commute	0.069	0.050	0.078	0.097
	[0.088]	[0.083]	[0.083]	[0.089]
Share of traffic delay in commute time	0.665***	0.628***	0.264**	0.360***
	[0.146]	[0.138]	[0.103]	[0.140]
Own automobiles	– 0.145	– 0.162	– 0.155**	– 0.140**
	[0.108]	[0.124]	[0.061]	[0.066]
No transit access within 10 min walk	0.048	0.044	– 0.084	– 0.052
	[0.142]	[0.138]	[0.120]	[0.117]
***Personal health consideration***				
Having children (5 or younger) with respiratory diseases in past 2 weeks	0.425*** [0.098]	0.391*** [0.085]	0.252** [0.107]	0.300*** [0.111]
***Civic Engagement***				
Voted in last presidential election		0.108 [0.140]	0.068 [0.099]	0.065 [0.115]
Household member actively participates in local institutions to improve neighborhood		0.217 [0.144]	0.036 [0.062]	0.048 [0.077]
***City fixed effects***				
Buenos Aires			(ref.)	
La Paz			1.475*** [0.043]	
Sao Paulo			0.876*** [0.036]	
Fortaleza			0.662*** [0.021]	
Bogota			0.743*** [0.067]	
Quito			1.126*** [0.044]	
Lima			1.578*** [0.044]	
Montevideo			– 0.159*** [0.020]	
Caracas			—0.179*** [0.037]	
Panama City			0.774*** [0.085]	
Mexico City			– 0.024 [0.051]	
***City-specific factors***				
GDP per capita (1000 USD)				– 0.041 [0.031]
Income Gini coefficient				2.253** [1.014]
City population (million)				– 0.039 [0.025]
Annual median PM2.5 around center (ug/m3)				0.059*** [0.013]
City has driving restrictions (1 = yes)				0.586*** [0.188]
***Personal socio-economic factors***	No	Yes	Yes	Yes
ρ^2^	0.016	0.023	0.079	0.064
Correctly classified	58.1%	58.6%	64.9%	62.1%

Note: Dependent variable = 1 if supporting either congestion pricing or driving restrictions, and 0 otherwise. Personal socio-economic factors include level of education, gender, age, age squared, living with spouse, having children, employment status, and home ownership. Model constants are not shown. Robust standard errors clustered at the city-level are in brackets. *, **, *** indicate statistical significance at 90%, 95%, and 99% levels of confidence, respectively. ρ^2^ measures the relative improvement in log likelihood of the full model over an intercept-only model. “Correctly classified” measures the percentage that the logistic regression model is able to predict the observed choices.

**Table 3:** Logistic regressions of support for congestion pricing and for driving restrictions (N = 8178).

	(5) Pricing city fixed effects	(6) Pricing city-specific factors	(7) Restrictions city fixed effects	(8) Restrictions city-specific factors
***Personal transportation factors***				
Used private automobiles in commute	– 0.383***	–0.384***	– 0.508***	– 0.514***
	[0.116]	[0.110]	[0.100]	[0.103]
Used rail or BRT in commute	0.014	0.043	0.048	0.065
	[0.051]	[0.072]	[0.098]	[0.096]
Used bus/taxi/informal transit in commute	0.097	0.127*	0.017	0.032
	[0.077]	[0.073]	[0.079]	[0.082]
Share of traffic delay in commute time	0.193	0.227	0.280***	0.325***
	[0.185]	[0.206]	[0.082]	[0.095]
Own automobiles	– 0.239***	–0.224***	– 0.160***	– 0.159***
	[0.067]	[0.069]	[0.058]	[0.061]
No transit access within 10 mins’ walk	– 0.074	– 0.040	– 0.120	– 0.088
	[0.077]	[0.078]	[0.116]	[0.116]
***Personal health consideration***				
Having children (5 or younger) with respiratory diseases in past 2 weeks	0.229** [0.107]	0.260** [0.113]	0.198** [0.086]	0.234*** [0.082]
***Civic Engagement***				
Voted in last presidential election	0.010 [0.104]	0.011 [0.120]	0.086 [0.073]	0.081 [0.076]
Household member actively participates in local institutions to improve neighborhood	– 0.008 [0.063]	0.016 [0.075]	0.079 [0.068]	0.098 [0.069]
***City fixed effects***				
Buenos Aires	(ref.)		(ref.)	
La Paz	0.780***		1.164***	
	[0.052]		[0.034]	
Sao Paulo	0.157***		0.765***	
	[0.031]		[0.033]	
Fortaleza	0.036		0.628***	
	[0.023]		[0.021]	
Bogota	0.126**		0.599***	
	[0.057]		[0.063]	
Quito	0.539***		0.894***	
	[0.051]		[0.038]	
Lima	1.000***		1.134***	
	[0.047]		[0.038]	
Montevideo	– 0.521***		– 0.229***	
	[0.022]		[0.019]	
Caracas	– 0.066*		– 0.345***	
	[0.036]		[0.035]	
Panama City	0.326***		0.316***	
	[0.071]		[0.073]	
Mexico City	– 0.166***		– 0.227***	
	[0.042]		[0.048]	
***City-specific factors***				
GDP per capita 1000 USD)		–0.053**		– 0.032
		[0.025]		[0.021]
Income Gini coefficient		– 0.313		3.150***
		[0.768]		[0.903]
City population (million)		– 0.008		– 0.033
		[0.021]		[0.020]
Annual median PM2.5 around center (μg/m^3^)		0.032***		0.053***
		[0.009]		[0.010]
City has driving restrictions (1 = yes)		0.221*		0.469***
		[0.130]		[0.149]
***Personal socio-economic factors***	Yes	Yes	Yes	Yes
ρ^2^	0.044	0.035	0.066	0.057
Correctly classified	65.9%	65.8%	63.7%	62.4%

Note: Dependent variable = 1 if supporting either congestion pricing or driving restrictions, and 0 otherwise. Personal socio-economic factors include level of education, gender, age, age squared, living with spouse, having children, employment status, and home ownership. Model constants are not shown. Robust standard errors clustered at the city-level are in brackets. *, **, *** indicate statistical significance at 90%, 95%, and 99% levels of confidence, respectively. ρ^2^ measures the relative improvement in log likelihood of the full model over an intercept-only model. “Correctly classified” measures the percentage that the logistic regression model is able to predict the observed choices.
