# Supplementary material for: Support for market-based and command-and-control congestion relief policies in Latin American cities: Effects of mobility, environmental health, and city-level factors
Source: Transp Res Part A Policy Pract. Author manuscript; Available in PMC 2021 Jul 21. (PMC7611337; doi:10.1016/j.tra.2020.12.004)
Supplement: Appendix A [file EMS129599-supplement-Appendix_A.pdf]

Appendix A

See Table A1

Table A1
Comparing individuals in and out of the study sample.

	In sample		Out of sample		Difference (in - out)	
	**mean**	**N**	**mean**	**N**	**diff**	**p-value**
Congestion relief policy support						
Support congestion pricing	0.36	8178	0.31	4212	0.05	0.000
Support driving restrictions	0.50	8178	0.47	4304	0.03	0.003
Support either pricing or ban	0.56	8178	0.52	4091	0.04	0.000
Personal transportation factors						
Used private automobiles in commute[±]	0.14	8178	0.12	3762	0.02	0.010
Used rail or BRT in commute[±]	0.19	8178	0.20	3762	−0.01	0.699
Used bus/taxi/informal transit in commute[±]	0.50	8178	0.52	3762	−0.02	0.056
Share of traffic delay in commute time	0.28	8178	0.28	3073	−0.01	0.263
Own automobiles	0.33	8178	0.30	4235	0.03	0.000
No transit access within 10 mins' walk	0.14	8178	0.11	2121	0.03	0.001
Personal health consideration						
Having children (5 or younger) with respiratory diseases in past 2 weeks	0.07	8178	0.06	4727	0.01	0.158
Civic engagement						
Voted in last presidential election	0.83	8178	0.83	4608	0.00	0.663
Household member actively participates in local institutions to improve neighbor	0.17	8178	0.18	4714	−0.01	0.057
Socio-economic characteristics						
Education						
Less than High School	0.39	8178	0.50	4715	−0.10	0.000
High school/some college	0.50	8178	0.44	4715	0.07	0.000
College or higher	0.11	8178	0.07	4715	0.04	0.000
Female	0.51	8178	0.58	4727	−0.07	0.000
Age (years)	37.10	8178	37.66	4726	−0.57	0.008
Living with partner	0.59	8178	0.62	4630	−0.03	0.003
Household with children	0.73	8178	0.74	4713	−0.01	0.141
Employed	0.67	8178	0.55	4618	0.12	0.000
Homeowner	0.67	8178	0.68	4638	−0.01	0.093

[±] Percentages do not add to 100 because categories are not mutually exclusive. Respondents selected all modes used.
